# 

**Published:** 1877-07-20

**Authors:** 


### CONTENTS.

ORIGINAL AND SELECTED ARTICLES—

Cincho-Quinine. By Ferdinand King. M D., Ph.G., Atlanta, Ga.................... 167
Advance in Pharmaceutical and Chemical Science. By Wm. A. Green, M.D., Macon, Ga. 170
Hemorrhagic Malarial Fever. By L. A. Guild, M.D., of Georgia.................... 172
The Wet Blanket Pack in Dysmenorrhea, Neuralgia, etc. By B. H. Washington, M.D., Augusta Ga.................... 174
Yerba Santa (Eriodyction Glutinosium.) By Dr. Gabel, Aurora, Ill.................. 176
On Constipation. By Wm. H. Thompson, M D., New York City.................... 177

ABSTRACTS AND GLEANINGS—

Indigestion; Constipation; Infantile Diarrhœa; Vomiting in Cholera Infantum, 180. Black Haw in Abortion and Dysmenorrhœa, 181 Gentiana Quinqueflora; Balsam of Tolu as an Application to Wounds, 182. Bilious Attacks: Use of the Obstetrical Forceps: Anæsthesia Produced by a Hypodermic Injection of Bromohydrate of Quinine; Treatment of Spasm of the Glottis, 183. Celluloid; Who Discovered Anæsthesia, 184. The feeding of Infants. Albumen in the Treatment of Consumption; Singular Property of the Tomato Leaf, 185. Varices of the Leg; Impaction of Gall-Stones; Administering Iodine Through a Nurse, 186. Dietetic Preparations, 187. Neuralgia of Female Eurethra; For Eczema; Lacto-Phosphate of Lime as a Tooth-Filling, 188.

PRACTICAL NOTES—

Perineal Abscess and Fistula of Urethra; Worms, 188. Hypodermic Doses; Jaborandi in Dropsy; Salacine for Chills; Congestive Chill; Iodoform Pencils, 189. Subnitrate of Bismuth in Diarrhœa; Bismuth and Pepsin in Diarrhœa; Simple Diarrhœa; Aromatic Syrup of Galls; Granulated Lids; Prof. Dowell's Ague Pill; Obstinate Intermittent; Prescription for Rheumatism; For Nervous Debility, 190. Cerebro-Spinal Meningetis: Visical Catarrh; Dysentery; Dialyzed Iron; Citrate of Caffien in Headache; To Abort Pneumonia: Chloral Cream; Chloral Emulsion; For After-pains; For Gonorhœa; For Baldness and Falling of Hair; Laxative for Piles, 191. Opium Modified: Suppression of Menses; In Hysterical Dysmenorrhœa; Colic, 192. Alhtura Wine; Benzoic Acid in Cystitis; Phosphorus, 193.

SCIENTIFIC ITEMS—

Sunlight; Plant Anæsthesia; New Functions of the Liver; The Microscope; Still Burning; Air in the Ground; Vitality of Snails; Antihydropin; Meteoric Stone, 193.

EDITORIAL AND MISCELLANEOUS—

Special Notice What's the Matter; Where to Send our Students; A Rabbi Speaks to a Medical Class; Benzoic Acid in Cystitis, 194. Electrolysis; Sawyer's Forceps; Uterine Dilator; Philadelphia Medicines for Egypt and Italy; Something New; The American Medical Association; Specific Tax on Physicians; Scientific Association at Nashville, 195. Books and Pamphlets Received; Merrell, Thorp & Loyd; Iron and Alum Mass; Cinquo-Quinine; Drs. Keller & Fox, 196.

